# Intramural left ventricular posterior superior process premature ventricular complex ablation with intracardiac echocardiography

**DOI:** 10.1016/j.ipej.2026.06.006

**Published:** 2026-06-18

**Authors:** Kunaraj Perumalu, Xuan Ming Pung, Eric Tien Siang Lim, Chi Keong Ching

**Affiliations:** Department of Cardiology, National Heart Centre Singapore, Singapore

## Abstract

Intramural premature ventricular complexes (PVCs) are notoriously challenging during catheter ablation due to the depth of the arrhythmogenic substrate. We present a case utilizing intracardiac echocardiography (ICE) integrated with 3D electroanatomical mapping to ablate an intramural PVC in a patient with methamphetamine-associated cardiomyopathy. A 22-year-old female with a history of stimulant use disorder presented with debilitating palpitations and a 46% PVC burden. PVCs originating from the posterior superior process of the left ventricle (PSP-LV) were successfully targeted under ICE guidance. There were no arrhythmia recurrences during follow-up, highlighting ICE as an invaluable tool for mapping and ablating PSP-LV origin PVCs.

**Learning Points**

• Given their specialized anatomical origin, intramural PVCs originating from the PSP-LV may require multichamber mapping during an electrophysiology procedure to ensure accurate localization.
• Intracardiac echocardiography provides real-time visualization and lesion depth monitoring during radiofrequency ablation
• Intracardiac echocardiography improves outcomes in complex intramural ventricular arrhythmias by facilitating precise catheter-tissue contact and identifying anatomical barriers. This approach is critical for targeting intramural arrhythmogenic substrates and ensuring therapeutic success.
• Ablating PSP-LV and intramural PVCs requires a highly structured approach. Furthermore, integrating emerging ablation modalities is essential to achieve favorable long-term outcomes.

## 1 Introduction

The PSP-LV constitutes the inferoposterior aspect of the basal left ventricle. Situated between the basal right and left ventricles, this region lies directly adjacent to the inferomedial right atrium. Because of this complex, central anatomy, PVCs originating from the PSP-LV can be successfully mapped and ablated from any of the three surrounding cardiac chambers: the right atrium, right ventricle, or left ventricle.

## 2 Case report

**Fig. 1:**
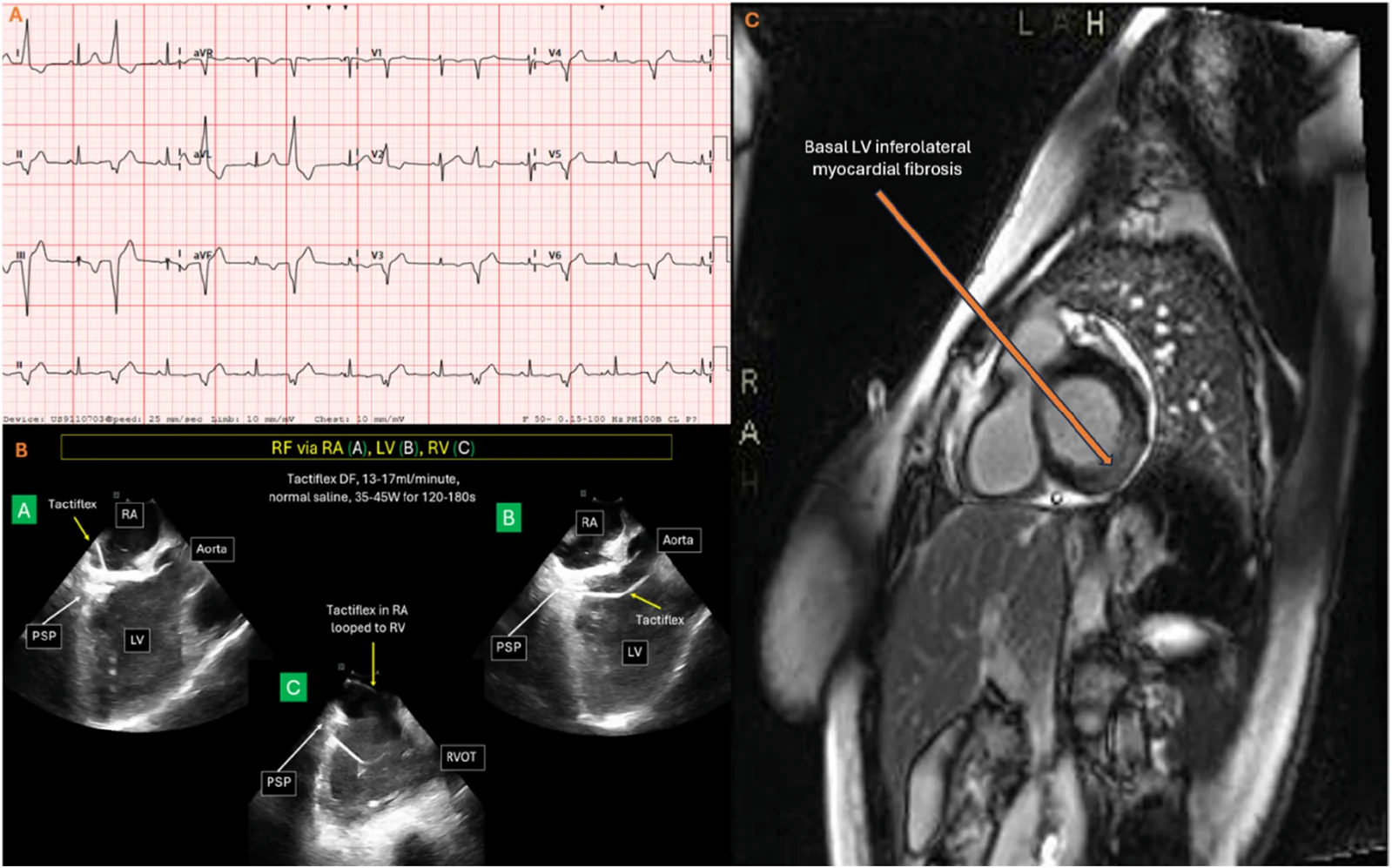
**A.** Left superior axis deviation with ‘reversed V2 lead pattern break’ and LBBB morphology in Lead V1 and short QRS duration of 86 ms. **B**. ICE imaging during targeted radiofrequency ablation within all three adjacent cardiac chambers (RA, RV, and LV**)**. **C.** Cardiac MRI with late gadolinium enhancement demonstrating myocardial fibrosis at the basal LV inferolateral region.

**Fig. 2:**
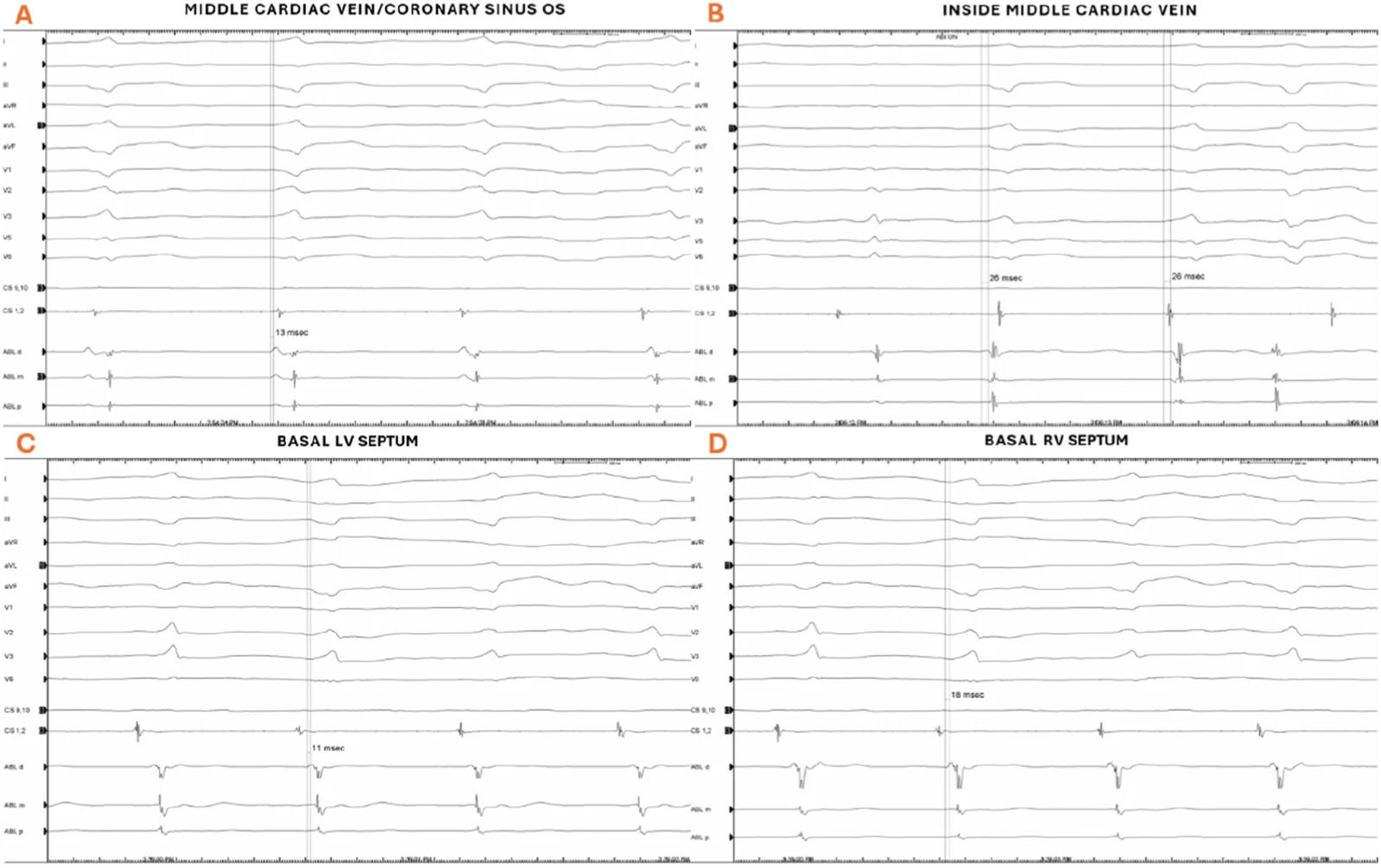
**A.** Electrogram signal at coronary sinus (CS); middle cardiac vein (MCV) ostium early by [-13 ms (milliseconds)] but appeared to be far field. **B.** Electrogram signal inside middle cardiac vein but local ventricular electrogram signal is late. **C.** Basal left ventricular septum prepotential electrogram signal was early by (−11 ms). **D.** The earliest prepotential electrogram signal was recorded at the basal right ventricular septum (−18 ms).

**Fig. 3:**
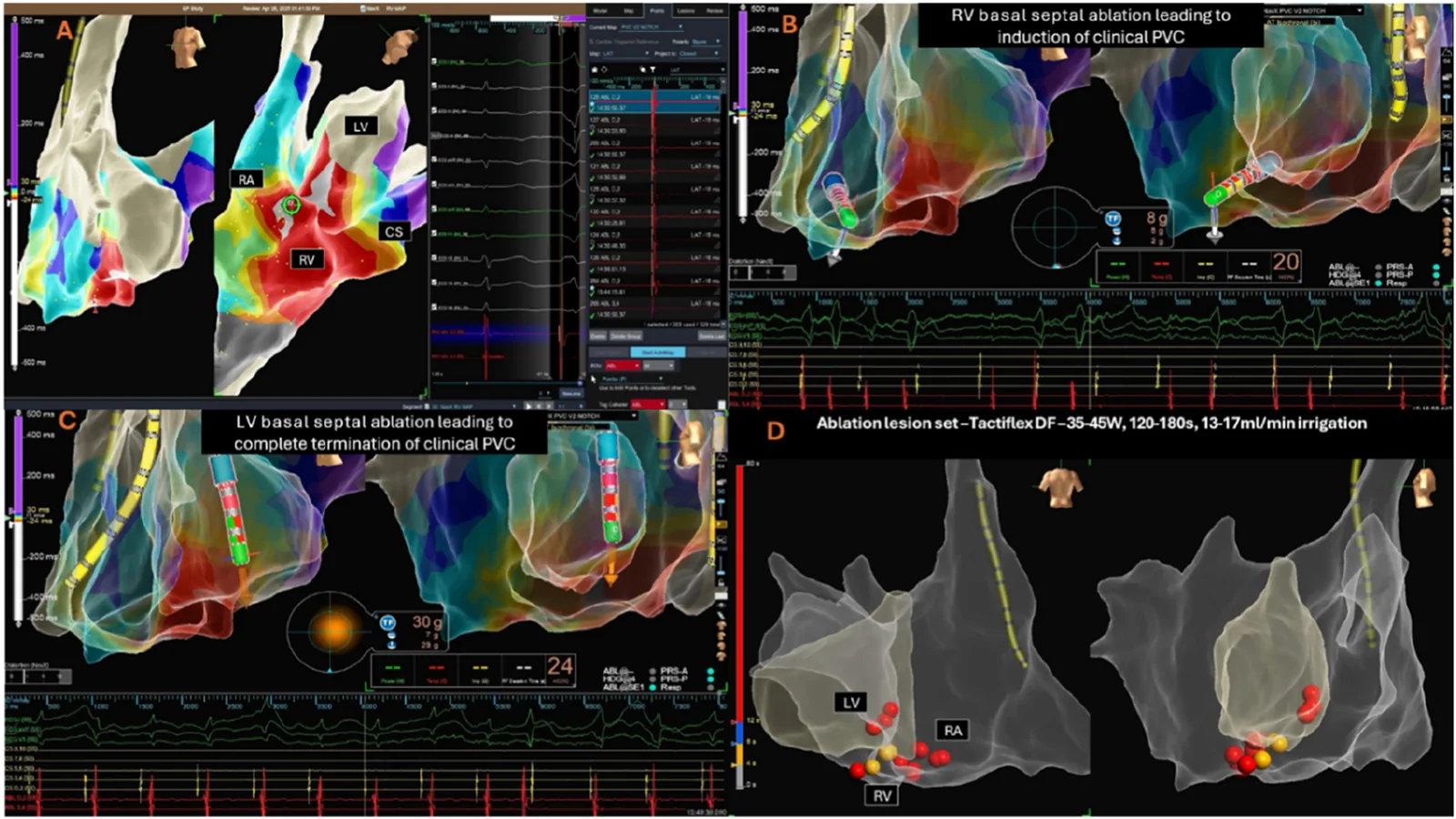
**A.** Local activation time (LAT) map of PSP-LV. **B.** Incessant PVCs were observed during basal right ventricle (RV) septal ablation. **C.** Catheter ablation of the basal inferoseptal process of the left ventricle resulted in permanent suppression of PVCs after 14.6 seconds. **D.** Complete suppression of PVCs achieved using an approach targeting three adjacent locations during ablation.

**Fig. 4:**
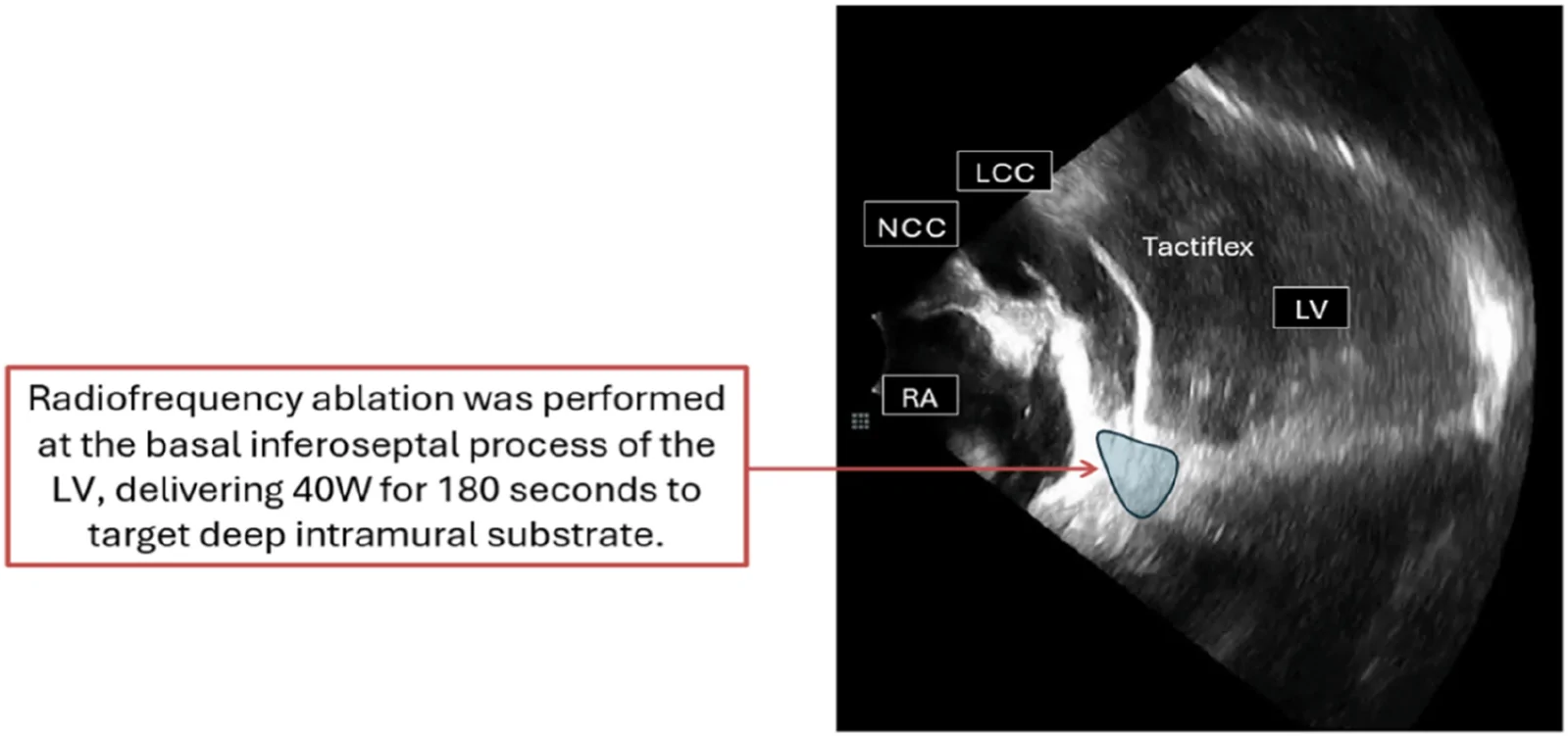
Intracardiac echocardiography demonstrating adjacent anatomical barriers and an area of hyperechogenicity at the ablation lesion site in the basal left ventricle.

A 22-year-old female with no known comorbidities presented with frequent palpitations associated with reduced exercise tolerance for 3 months. She had no other cardiovascular risk factors, family history of cardiac arrhythmias, or known comorbidities. She had a history of anxiety and stimulant use disorder related to amphetamine use. Her palpitations did not subside despite methamphetamine cessation. Two PVCs with similar QRS morphologies consistent with left ventricular basal posterior origin were recorded (Fig. 1). 24-hour Holter recorded PVC burden of 46%. Transthoracic echocardiogram left ventricular ejection fraction (LVEF) was 35%. Cardiac MRI showed mid-wall fibrosis of the basal inferolateral segment of the left ventricle with mid-wall myocardial late gadolinium enhancement (Fig. 1C). She underwent electrophysiology study. 3D electroanatomical activation mapping showed focal activation at the basal right and left ventricular septum – EGMs prepotential signals were earlier by 18 ms and 11 ms relative to PVC onset from the right and left sides respectively (Fig. 2). Coronary sinus and middle cardiac vein activation was late. Radiofrequency catheter ablation (RFA) was initially performed from the basal right ventricle as well as the anatomically adjacent right atrium, as the local activation map showed these were the earliest activation sites (Fig. 3). However, the PVCs persisted, and a decision was made to ablate from the basal left ventricle, which led to permanent suppression of the clinical PVCs. RFA was performed using Tactiflex™ DF, Abbott, AM Plymouth, USA (contact force sensing open-irrigated ablation catheter) at 35 W to 45 W for 180 seconds using normal saline irrigation of 13 to 17 mL/min which led to complete suppression of PVCs (Fig. 4). The sandwich concept was applied in our case because initial RFA at the basal right ventricle and adjacent right atrium induced runs of clinical PVCs; however, complete suppression was not achieved. Therefore, finding early prepotential signals at the basal left ventricle prompted us to deliver the final RFA there, which completely suppressed the PVCs. Isoproterenol infusion up to 5 mcg/min post ablation showed no PVC recurrence. She remained asymptomatic with no recorded PVCs on 12-lead surface electrocardiogram at 2-month follow-up. At the 9-month follow-up, 24-h Holter monitoring showed no evidence of PVCs; additionally, a transthoracic echocardiogram demonstrated normalization of LVEF to 60%.

## 3 Discussion

[1]A 12-lead electrocardiogram with left bundle branch block (LBBB) pattern in lead V1, short QRS duration, ‘reverse V2 lead pattern break’ together with left superior axis deviation should raise suspicion for PVCs originating from the inferoseptal process of basal left ventricle, also known as the PSP-LV.

[2] The earliest electrogram signal during the electrophysiology study in this case was comparable between the basal septal right and left ventricles, suggesting the arrhythmic substrate originated intramurally. Pace mapping at the earliest activation site may yield a poor pace match due to this intramural origin.

[[3], [4], [5], [6]]Data concerning PVCs in this anatomical region are scarce, and their overall prevalence remains low. Despite this, studies indicate that ventricular arrhythmias originating from the PSP-LV exhibit higher recurrence rates and may feature multiple exit sites, precluding an established optimal ablation strategy.

Given the recalcitrant PVCs, we elected to ablate from the three adjacent cardiac chambers to maximize the chances of success.

[7]The chronic use of stimulant drugs as shown in our case may have contributed to depressed left ventricular function and predisposed the patient to PVCs. It is known that use of methamphetamines is associated with PVCs, possibly via catecholamine surge and overstimulation of the sympathetic nervous system. Methamphetamine triggers massive release of norepinephrine and dopamine, which stimulate beta-adrenergic receptors and further increase calcium levels within cardiac myocytes. The prolonged sympathetic overstimulation leads to direct cardiomyocyte damage which includes contraction band necrosis and accelerated apoptosis that eventually leads to fibrous tissue formation [8].Methamphetamine also disrupts calcium homeostasis within myocytes which leads to delayed afterdepolarizations triggering PVCs.

Methamphetamine causes significant intracellular calcium dysregulation by triggering an increase in cytosolic calcium levels via release from internal repositories, such as the endoplasmic or sarcoplasmic reticulum, and by upregulating the expression of membrane-bound L-type Ca2+ channels during chronic exposure [9].Excessive intracellular calcium is a primary driver of delayed afterdepolarizations which occur when surplus calcium is expelled from the cell via the Na^+^-Ca^2+^ exchanger creating an inward current that depolarizes the membrane. These delayed afterdepolarizations trigger PVCs when they reach the threshold for an action potential. Methamphetamines have also been shown to cause structural remodelling which includes mid-wall myocardial fibrosis as shown in the cardiac MRI in our case. Mid-wall fibrosis in the basal inferolateral segment is a recognized hallmark of methamphetamine-associated cardiomyopathy.

[[10], [11], [12]]The utilization of high-power, long-duration ablation (e.g., 40 W to 70 W for 60 to 90 seconds) for PSP-LV PVCs is an alternative approach to achieve deep tissue penetration via conductive heat transfer, provided the baseline impedance and contact force are optimized. However, prolonged applications carry a distinct risk of ‘steam pops' caused by deep intramural overheating, which increases the risk of cardiac perforation. This may inadvertently increase the risk of microbubble or microemboli formation. Good contact force mitigates some stability risks but amplifies localized resistive heating at the tip. Furthermore, applying ineffective RFA without successfully suppressing the clinical PVCs leads to local tissue edema. This edema increases the distance between the catheter tip and the deeper arrhythmogenic substrate, rendering subsequent ablation attempts less effective until the edema resolves. While high-power short-duration (HPSD) or very high-power short-duration (vHPSD) strategies (e.g., 45 W to 90 W for 4 to 15 seconds) shift the biophysical profile to rely almost exclusively on immediate resistive heating rather than conductive heating, they produce wider, shallower lesions that are often insufficient for reaching deep ventricular arrhythmias.

[13,14]Several strategies exist for treating complex, resistant intramural ventricular arrhythmias. One option is bipolar ablation using two catheters from opposite sides of the myocardial wall to ensure adequate transmural lesions. Studies suggest that a power setting of 30 W for longer durations (e.g., 120 s) yields the safest and most optimal lesion geometry, particularly when maintaining a parallel catheter configuration. Because RFA at higher powers (≥40 W) significantly elevates the risk of steam pops, careful monitoring of impedance dynamics is crucial to prevent these events. Currently, bipolar RFA is often employed as a bailout strategy or advanced adjunctive method for patients whose arrhythmias have proven refractory to standard unipolar ablation procedures. A recent 2025 large single-center study, titled “*Bipolar Catheter Ablation as a Key for Successful Treatment of Intramural Ventricular Arrhythmias: Results From the Bipolar-Kiel Study*” by Fabian Moser et al., demonstrated that bipolar ablation is safe and feasible in patients presenting with intramural arrhythmias. This approach can be utilized during the first procedure or following a previously failed ablation attempt. Furthermore, endo-epicardial bipolar ablation represents a highly useful tool for targeting intramural substrates.

[15,16]Additionally, chemical ablation of ventricular arrhythmias can be delivered via either a retrograde coronary venous or a transcoronary arterial route. This approach is particularly beneficial for targeting deep intramural substrates, such as the interventricular septum. Retrograde coronary venous ethanol ablation (RCVEA) is generally preferred over transcoronary ethanol ablation (TCEA), as TCEA carries a higher risk of myocardial infarction and coronary artery dissection.

When targeting the posterior superior process of the left ventricle via the transcoronary route, operators may utilize the posterior descending artery, the origin of which depends on coronary dominance. Furthermore, if coronary venous tributaries are inaccessible by standard mapping catheters, wire mapping serves as the optimal alternative. Target sites are identified by mapping the earliest presystolic signals and confirming a match with the clinical PVC. Once the optimal venous branch is isolated, an angioplasty balloon catheter is inflated to occlude the vein, followed by a slow infusion of ethanol (approximately 1 mL over 1 minute) to ablate the arrhythmogenic tissue.

While highly effective, procedural success is strictly dependent on favorable venous anatomy that directly supplies the target substrate. The technique remains technically challenging due to the high variability of the cardiac venous network, and risks include venous dissection or perforation leading to pericardial effusion. Nevertheless, contemporary literature, including a review by Adi Lador et al. titled “*Alcohol Ablation for Ventricular Tachycardia*”, demonstrates that RCVEA can achieve an acute success rate of up to 98% for these challenging ventricular arrhythmias.

[17]Alternative approaches to intramural ablation encompass irrigated needle ablation techniques. A clinical trial titled, “*Worldwide Experience With an Irrigated Needle Catheter for Ablation of Refractory Ventricular Arrhythmias: Final Report*” by Usha B Tedrow et al. showed acute successful abolishment of the targeted PVCs in 89% of patients. Furthermore, at 6 months of follow-up, 78% of patients achieved the clinical success threshold, maintaining a reduced PVC burden of less than 5000 counts over 24 hours. This procedure utilized a specialized catheter featuring a retractable 27-gauge end-hole needle, which was advanced 7 to 9 mm directly into the myocardium to deliver localized saline irrigation alongside radiofrequency energy. While irrigated needle ablation catheters can also create intramural lesions, they are not readily available in most centres.

[18,19]Whereas the use of Pulsed Field Ablation (PFA) in ventricular chambers, including the posterior superior process, represents an evolving frontier. Treatment in this area is typically performed off-label using focal contact-force sensing PFA catheters or as part of active clinical trials investigating VT and PVCs. Early cohort studies and case series show high acute success rates with minimal procedural complications, as shown in *Focal Pulsed Field Ablation for Premature Ventricular Contractions: A Multicenter Experience* by Domenico Giovanni Della Rocca et al.

Because cardiomyocytes possess a lower threshold for electroporation than other cells, PFA can selectively eliminate arrhythmogenic heart tissue while sparing nearby coronary arteries, veins, and nerves. Furthermore, PFA energy can overcome the insulating layers of epicardial fat and penetrate deeply into the thick basal ventricular myocardium, achieving transmural lesions that radiofrequency energy often fails to complete. In a study by Jim Hanssen et al., the application of PFA in a patient with failed RFA at the basal left ventricle showed a favorable response with the termination of PVCs. However, the off-label use of PFA in this region warrants more research, and systematic studies with regard to safety, efficacy, and long-term outcomes are needed.

[20,21]Finally, the use of a low-ionic irrigant solution, specifically half-normal saline (0.45% NaCl), is supported by existing literature for its ability to create deeper and larger lesions. The low-ionic content in the irrigant helps create a high impedance cloud around the electrode, simultaneously increasing current density at the tip-tissue interface, which enables the delivery of larger and deeper lesions.

To our knowledge, this is the first case report of a methamphetamine-induced cardiomyopathy associated with intramural PVCs from PSP-LV.

[22]The utilization of intracardiac echocardiography is beneficial in a number of ways: (i) it provides real-time, high-resolution imaging of the PSP-LV, (ii) it provides information regarding catheter contact, depth of ablation lesions and safety during high power long duration ablation, and (iii) minimizes need for fluoroscopy. The features make it ideal for ablation of intramural PVCs originating from the PSP-LV.

## 4 Conclusion

Ablation of intramural PVCs originating from the PSP-LV can be challenging. A comprehensive approach that includes understanding the anatomy of this region is needed to maximize ablation success.

## Patient consent

Informed consent for publication was obtained from the patient in accordance with COPE guidelines. The consent was obtained using the standard consent form approved by our institution.

## Disclosures

The authors have no conflict of interest to disclose.

## Authorship

All authors attest they meet the current ICMJE criteria for authorship.

## Ethics statement

All procedures were conducted in accordance with the ethical guidelines and regulations outlined in the Declaration of Helsinki.

## Funding

This research did not receive any specific grant from funding agencies in the public, commercial, or not-for-profit sectors.

## Declaration of competing interest

The authors declare that they have no known competing financial interests or personal relationships that could have appeared to influence the work reported in this paper.

## Data Availability

The data underlying this article will be shared upon reasonable request to the corresponding author.
